# Emergency Nursing-Care Patient Satisfaction Scale (Enpss): Development and Validation of a Patient Satisfaction Scale with Emergency Room Nursing

**DOI:** 10.3390/healthcare10030518

**Published:** 2022-03-12

**Authors:** Junpei Haruna, Naomi Minamoto, Mizue Shiromaru, Yukiko Taguchi, Natsuko Makino, Naoki Kanda, Hiromi Uchida

**Affiliations:** 1 Department of Intensive Care Medicine, School of Medicine, Sapporo Medical University, Sapporo 060-8543, Japan; 2 Department of Nursing, Sapporo City General Hospital, Sapporo 060-8604, Japan; m.naonene@gmail.com; 3 Department of Nursing, School of Health Sciences, Sapporo Medical University, Sapporo 060-8556, Japan; shiro.m@sapmed.ac.jp (M.S.); y.taguchi@sapmed.ac.jp (Y.T.); 4 Department of Nursing, Sapporo Medical University Hospital, Sapporo 060-8543, Japan; natsu626kyoro@hotmail.com (N.M.); sakuhiro0712@hotmail.co.jp (H.U.); 5 Department of Nursing & Social Services, Health Sciences University of Hokkaido, Tobetsu 061-0293, Japan; kanda@hoku-iryo-u.ac.jp

**Keywords:** emergency room, patient satisfaction scale, emergency nursing

## Abstract

This study aimed to develop and validate an emergency nursing-care patient satisfaction scale to measure patient satisfaction with emergency room (ER) nursing. Patient satisfaction scales for ER nursing have been validated without considering the perspectives of the healthcare system or cultural background of the country. Moreover, although nursing care is changing with COVID-19, no scale has been specifically designed to assess patient satisfaction with ER nursing. The study population included patients who visited five ERs in Japan (March to December 2021) (*n* = 135). The rating scales were provided to patients who visited the ER and gave consent, and the patients were asked to reply. In the process of validating the scale, exploratory and confirmatory factor analyses of the construct and criterion validity were conducted. The confirmatory factor analysis results showed a factorial structure consisting of four factors. The domain and summary scores demonstrated good-to-excellent internal reliability (Cronbach’s range = 0.81–0.89). This patient satisfaction scale was designed and validated from the perspective of the Japanese healthcare system and cultural backgrounds. This scale may be useful for developing assessments and interventions to improve patient satisfaction with ER nursing.

## 1. Introduction

Patient response to healthcare services is one of the best ways to obtain information about the quality of healthcare [1]. In particular, patient satisfaction is an important indicator for evaluating service quality and improving healthcare services, and is a commonly used and valid indicator [2,3]. The field of medicine is divided into specialties, and specialized treatment and care are provided in each field. In measuring patient satisfaction, the development of satisfaction scales for each specialty was reported to help deal with specific problems [4]. In nursing, patient satisfaction is defined as the extent of the gap between patient expectations of ideal nursing care and their perception of the nursing care received [5]. We consider it important to use information obtained from patient satisfaction to improve care and enhance the quality of healthcare services.

Emergency nurses have multiple challenging responsibilities, including dealing with overstressed patients and their relatives, homeless and mentally ill patients, and victims [6]. In addition, emergency nurses play several roles, including triage [7], first aid [7], and early recognition of critically ill patients [8]. In recent years, emergency care has become more urgent worldwide due to the increasing severity of coronavirus disease 2019 (COVID-19) patients, increasing the burden on emergency nurses [9]. However, even during the COVID-19 pandemic, rapid and appropriate responses to emergency patients are required.

Various models have been used to measure satisfaction with emergency care [4,10,11]. The Consumer Emergency Care Satisfaction Scale (CECSS) is one of the most widely used patient satisfaction surveys for emergency patients in many countries [10]. Many studies using existing satisfaction scales have been reported for triage nurses [12,13] and have been conducted in a variety of geographic areas, ranging from urban to rural areas [14]. Factors associated with patient satisfaction with emergency care have been consistently reported in previous studies to be related to staff attitudes, explanations to patients, communication, time spent waiting in the emergency room (ER) [15,16], and environmental factors in the ER [17,18]. Satisfaction with emergency care has also been reported to be related to organizational culture [19] and healthcare professional burnout [20,21]. Therefore, measuring patient satisfaction is of great importance to healthcare professionals in the ER setting, as patient satisfaction measures can be used to identify weaknesses in their respective facilities.

However, patient satisfaction with the healthcare provided is influenced by several characteristics, such as the cultural background of the country or region [22], race [23], the healthcare system [24], the insurance system [25], the educational system for nurses [26], and the economic situation of the country [27]. Even though patient satisfaction is used in many countries, these factors limit the ability to measure patient satisfaction that reflects country-specific characteristics. In addition, a systematic review of patient satisfaction surveys reported that there is no gold standard for measuring patient satisfaction [28], and that it is necessary to develop instruments that are appropriate for the healthcare system of each country and other factors.

There are three levels of emergency hospital designation in Japan: “primary” for patients who can be managed as outpatients, “secondary” for patients who need to be hospitalized, and “tertiary” for patients who need to be managed in an intensive care unit (ICU) [29]. Japan has one of the most aged populations in the world [30], and the number of patients visiting the ER is on the rise because of the COVID-19 pandemic [31]. Moreover, Japan has had a universal health coverage system since 1961, providing equal access to healthcare for all citizens at all times [32]. Furthermore, in terms of nursing specialization, although there are specialists, such as certified nurses and certified nurse specialists, they account for approximately 1% of all nurses, and the current situation is that there is insufficient training of specialists [33]. However, the current situation in Japan differs from that in other countries. Therefore, the patient satisfaction scale for emergency nurses currently in use does not necessarily match the assessment in Japan. Furthermore, the recent COVID-19 pandemic has changed patient satisfaction in the ER. Patient satisfaction with ER staff tended to be high at the beginning of the COVID-19 pandemic. This suggests an overall appreciation for care during the early stages of the COVID-19 pandemic [34]. Furthermore, patient satisfaction with the ER has decreased during the COVID-19 pandemic due to increased workloads, such as infection control measures [15]. In addition, the COVID-19 pandemic would lead to overcrowding in the ER and lower patient satisfaction [35]. However, no scale has been developed to measure patient satisfaction with emergency nurses in Japan to the best of our knowledge. This study aimed to develop and validate a patient satisfaction scale to measure patient satisfaction with ER nursing.

## 2. Materials and Methods

### 2.1. Study Design

This study had the following steps to achieve its objectives: (1) Developing items to measure patient satisfaction with ER nursing, (2) examining content validity, (3) selecting items, and (4) examining construct validity.

(1) Development of items to measure patient satisfaction with ER nursing

In this phase, we generated various items according to the different categories established by the CECSS [10] and the Patient Satisfaction Questionnaire Short Form (PSQ-18) [2]. The items were elaborated and written through four consensus meetings with consultations from experts in developing the measuring instruments. The research team consisted of eight experts: two certified nurses in emergency nursing, one certified nurse in operation nursing, three certified nurse specialists in critical care nursing, and a university professor of nursing in critical care [36]. The first version of the questionnaire was developed on the basis of the four dimensions of satisfactory care, with 34 items distributed among the four factors. Additionally, we conducted our review using the keywords “emergency care”, “emergency nursing”, and “patient satisfaction” and searched CINAHL, PubMed, Medline, and other databases in the field. As a result, 12 items were identified, and 46 items in six categories were generated.

(2) Content validation

Previous studies have reported that it is desirable to reflect the opinions of experts in the field as well as patients and healthy people who are involved in the content validation process [37]. Content validation of the first questionnaire version was conducted by an expert panel of 10 healthy individuals who had visited an ER and 9 certified nurses in emergency nursing [38] (Table A1). We sent questionnaires to the panel of 19 people and asked them whether the survey items were valid. The survey items were assessed on a 4-point Likert scale ranging from 1 (“not at all important”) to 4 (“very important”). We also asked about repetition, incomprehensibility, and ease of answering. Following the method proposed by Davis, the item-level content validity index (I-CVI) was calculated by dividing the number of experts who gave a rating of three or four for each item by the total number of experts [39]. Items with an I-CVI lower than 0.78 were eliminated [40]. Numerical codes were assigned to the completed forms to ensure confidentiality and anonymity of the questionnaires. The final version of the questionnaire was established after deleting five items on the basis of content validation research analysis and the results obtained from various consensus meetings between the research team and expert advisors. The 46 items included in the first version were reduced to 39 in the final version of the emergency nursing-care patient satisfaction scale (ENPSS).

(3) Selecting items

Participants

The emergency medical care system in Japan is classified into three categories: primary emergency facilities that mainly treat patients who can return home without the need for hospitalization, secondary emergency facilities that mainly treat critically ill patients who require hospitalization, and life-saving emergency centers that treat critically ill patients who require advanced treatment [41]. In this study, patients who visited primary and secondary emergency facilities were included. The patients fulfilled the following criteria to participate in the validation study:

1. Age ≥ 18 years at the time of recruitment;
2. Capable of providing consent;
3. Ability to answer questionnaires;
4. Cognitive and physical capacity to complete self-administered questionnaires without the need for a proxy.

Participants were recruited from the emergency departments of five facilities in Japan between March 2021 and December 2021. The questionnaire was distributed to patients who visited the ER and met the above criteria. The research collaborator, an emergency nurse, asked each respondent to complete the questionnaire individually.

The sample size was targeted at >100 participants based on the COnsensus-based Standards for the selection of health Measurement Instruments (COSMIN) checklist, a guideline for scale development [37,42].

Survey components

The survey consists of four components. The first was a questionnaire on individual and institutional characteristics. The second part consisted of the number of visits to the ER, the time of day when they visited the ER, and the length of time they waited in the ER. The third component consisted of the 6-item EuroQol 5 dimensions 5-level (EQ-5D-5L) [43,44] to test criterion-related validity and the intensity of distress during the ER visit and overall satisfaction in the ER. The fourth component consisted of a satisfaction survey of the nurses in the ER. 

Instruments

The EQ-5D-5L is a validated and standardized instrument that measures health-related quality of life (QOL) [43,44]. A Japanese version of the EQ-5D-5L is available [45]. The EQ-5D-5L consists of the following five dimensions: mobility, self-care, usual activities, pain/discomfort, and anxiety/depression. Each dimension has five levels: no problems, slight problems, moderate problems, severe problems, and extreme problems. Health status is represented in 3125 combinations, and each combination of answers can be converted into a QOL score, ranging from 0 (death) to 1 (perfect health), according to a Japanese value set [45]. We compared the scores from our study with those of a previously reported Japanese norm [46]. The EQ-5D-5L also uses a visual analog scale (VAS) ranging from 0 to 100, where 0 represents the worst imaginable health and 100 represents the best imaginable health.

To determine the content of the questions related to patient satisfaction with ER nursing, we first examined the available information from previous studies [47,48,49,50]. Second, we extracted content related to satisfaction with ER nursing. Third, on the basis of these contents, five certified nurses were interviewed. On the basis of these results, the following four items were adopted in this study, and each question was measured using the VAS with “strongly agree” as 100 and “disagree” as 0.

1. Confidence in the ER’s physician;
2. Satisfaction with the response of ER’s physician;
3. Intensity of distress at the time of ER visit;
4. Satisfaction with the outcome of treatment in the ER.

For each of the patient satisfaction surveys for nurses in the ER, respondents rated their level of agreement on a standard five-point Likert scale (0 = “not applicable”, 1 = “strongly disagree”, 2 = “slightly disagree”, 3 = “neutral”, 4 = “slightly agree”, and 5 = “strongly agree”).

### 2.2. Statistical Analysis

Descriptive statistics were derived for the analysis. Categorical data were expressed as numbers and percentages. 

We examined some of the questionnaire items for possible exclusion according to the following criteria: items with a 20% rate or higher of “not applicable” [51] and items with an average score of 4.5, 1.5, or lower for each item. Furthermore, one of the items with a correlation coefficient of 0.7 or higher for each item was eliminated [52].

Exploratory factor analysis (EFA) using promax rotation and maximum likelihood extraction methods was conducted to determine the number and type of factors from 38 of the 39 survey items, excluding the question on overall satisfaction. EFA was conducted on the complete data for all 38 items at baseline. The factor solution from the EFA was based on the magnitude of the factor loadings for each item. On the basis of standard psychometric criteria, items with factor loadings of less than 0.35 were eliminated. The researchers assessed whether the elimination or retention of specific items was meaningful for assessing patient satisfaction.

On the basis of the results of the EFA, a confirmatory factor analysis (CFA) was used to evaluate factor solutions. The goal of the CFA was to evaluate the model fit of the factor structure using the root mean square error of approximation (RMSEA: where <0.09 is considered acceptable and <0.06 is considered excellent) and comparative fit index (CFI: where >0.9 is considered acceptable). As a result, we named each factor that represented various aspects of patient satisfaction with nursing in the ER.

The internal consistency reliability of the ENPSS was assessed using Cronbach’s alpha. The estimates of reliability should exceed 0.70 (0.7 ≤ α < 0.8 is acceptable, 0.8 ≤ α < 0.9 is good, and 0.9 ≤ α is excellent) [53,54]. Construct validity was assessed using Pearson correlations of the EFA-yielded domains and the ENPSS summary score with the five validated questionnaires, namely, EQ-5D-5L (using the VAS), anxiety on EQ-5D-5L, distress at ER visit (VAS), confidence in physician (VAS), and satisfaction with treatment (VAS). We hypothesized that the ENPSS would correlate more with QOL [55] since satisfaction with treatment is associated with QOL.

Only questionnaires with complete data were included in the analysis, and there was no imputation of missing data. Statistical significance was set at *p* ≤ 0.05 (two-sided). Statistical analyses were performed using SPSS Statistics version 27 (IBM Corp., Armonk, NY, USA) and JMP Pro software version 15 (SAS Institute Inc., Cary, NC, USA). 

### 2.3. Ethical Considerations

The protocol for this research project was approved by a suitably constituted Ethics Committee of Sapporo Medical University and conformed to the provisions of the Declaration of Helsinki, Approval No. 1-2-51. Informed consent was obtained from all the respondents. Participants were informed of the purpose and length of the survey, and their participation was voluntary. Consent was obtained from respondents by checking the box on the front page of the questionnaires that they understood the research explanation and agreed to participate according to Institutional Review Board recommendations.

## 3. Results

### 3.1. Population

A total of 127 respondent surveys were included in the final analysis after excluding eight surveys with missing data. The characteristics of respondents are presented in Table 1. Patients who were still employed accounted for 66.1%, and those with underlying diseases accounted for 63.0% of the total. Fifty-two percent of the patients visited the ER during the day, and 40.2% visited the ER for the first time.

### 3.2. Selecting Items

A total of 127 participants (92%) had complete data on the ENPSS and constituted the population used for factor analysis; this was sufficient for previous EFA studies [37,42,56,57]. 

First, of the 38 question items, 4 items of which more than 20% were answered as not applicable were deleted. There were 31 pairs with correlation coefficients greater than 0.7. We eliminated one of the items of the pair, and 14 items were excluded. Second, factor analysis was conducted using the maximum likelihood method. In the EFA using promax rotation, one item with a factor loading of less than 0.35 was removed, and finally, 20 items were selected (Table 2). 

**Figure 1:**
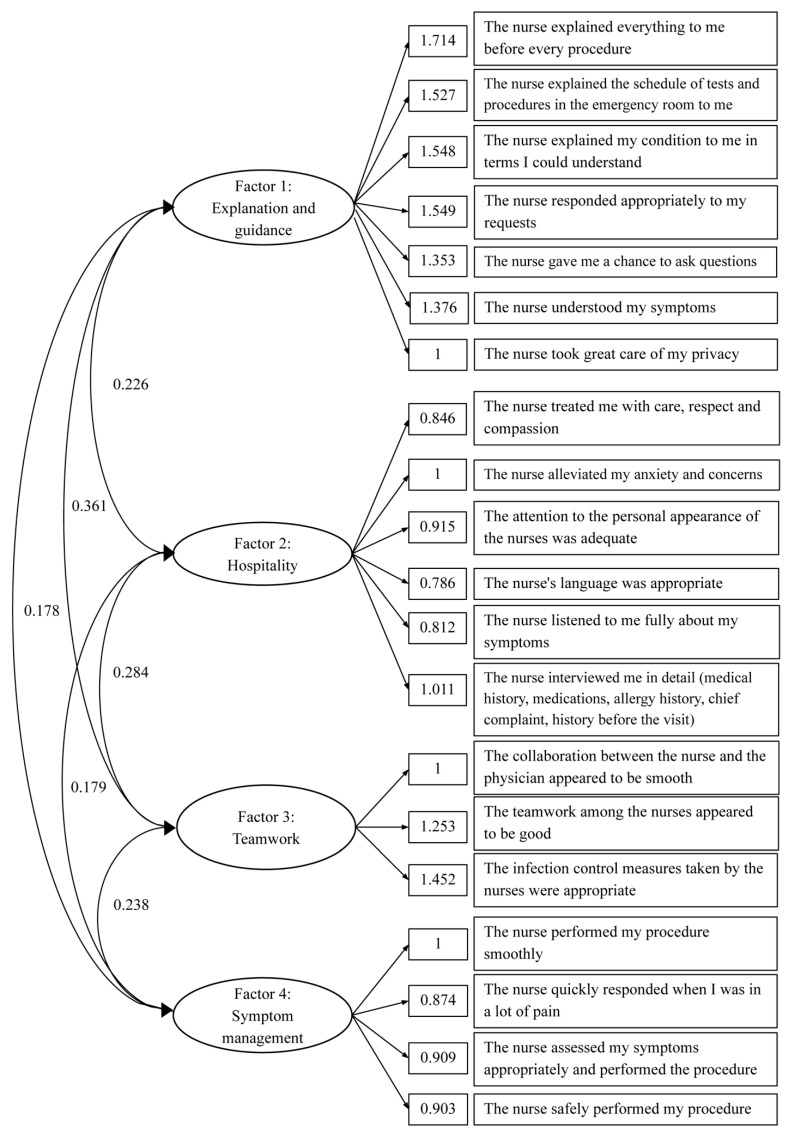
Confirmatory factor analysis.

The EFA yielded 20 solution items loaded into four factors representing four domains: explanation and response (seven items), hospitality (six items), teamwork (three items), and symptom management (four items). The global satisfaction item was not included in the EFA, which constitutes the ENPSS-21 in English version (see Appendix B) and Japanese version (see Appendix C). This 21-item questionnaire’s factor structure CFA (Figure 1) showed an acceptable fit: RMSEA = 0.1 (90% confidence interval = 0.08–0.11) and CFI = 0.9. EFA, exploratory factor analysis; ENPSS, emergency nursing-care patient satisfaction scale; CFA, confirmatory factor analysis; RMSEA, root mean square error of approximation; CFI, comparative fit index.

### 3.3. Internal Consistency Reliability

The internal consistency reliability (Cronbach’s alpha) of the four domains of the ENPSS-21 ranged from 0.81 to 0.89 (Table 3), corresponding to good internal reliability.

### 3.4. Construct Validity

**Figure 2:**
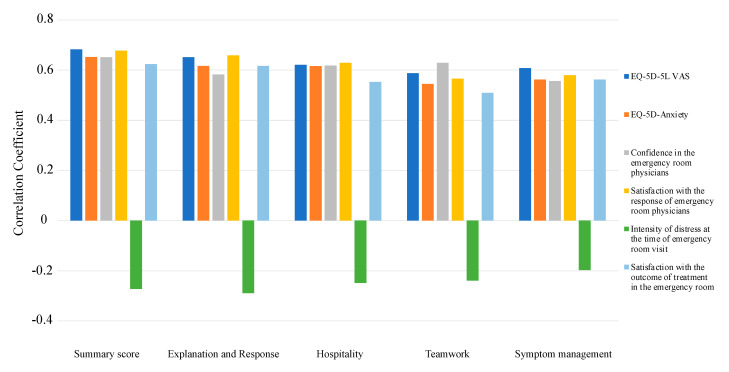
Construct validity of the 19-item Emergency Nursing-Care Patient Satisfaction Questionnaire (ENPSS-21).

The correlations of the four domain scores of the ENPSS-21 showed that the EQ-5D-5L VAS (0.59–0.65, *p* < 0.01), EQ-5D-5L Anxiety (0.55–0.62, *p* < 0.01), confidence in the ER physicians (0.56–0.63, *p* < 0.01), satisfaction with the response of ER physicians (0.57–0.66, *p* < 0.01), and satisfaction with the outcome of treatment in the ER (0.51–0.62, *p* < 0.01) were in the moderate to high range. The correlation was lower for the intensity of distress at the time of the ER visit (−0.2–−0.29, *p* < 0.01). As hypothesized, the summary score of ENPSS-21 showed the strongest correlation with EQ-5D-5L (0.68) and satisfaction with the response of the ER physician (0.68). (Figure 2 and Table 4).

## 4. Discussion

In this study, we developed a patient satisfaction scale focused on ER nursing, confirmed its validity and reliability, and concluded that it could be used in clinical practice. To the best of our knowledge, there are no reports on developing a patient satisfaction scale that focuses on ER nursing in Japan. 

The factor analysis results were composed of four domains: “explanation and response”, “hospitality”, ”teamwork”, and “symptom management”. This multidimensional structure is consistent with many reports on patient satisfaction analysis [2,10,24,58,59]. Furthermore, the specific dimensions obtained in this study are similar to those found in other scales [10,60], which we consider partially supportive of the construct validity of this tool. 

A limitation when measuring patient satisfaction is that psychometric properties may not be reflected because cultural factors from different countries and regions are not adequately taken into account [61]. Translation of existing patient satisfaction measures, such as CECSS and others, may lead to differences in the perception of quality of care from the patient’s perspective due to cross-cultural differences [62]. Because of the specific situation in ERs, where patients are more urgent than in general wards and require a variety of responses, a specific scale consistent with the culture of the country is considered essential. Therefore, a patient satisfaction scale in the ER that takes into account the Japanese cultural context was needed. 

The ENPSS-21 domain “explanation and response” includes nurse–patient communication and provision of information. In order for the nurse to meet the needs of the patient, a natural and constructive relationship must be established [63]. Nurses can then provide counseling and guidance to patients to improve patient satisfaction [64]. In the ER, providing information and communication to patients is also an important factor in facilitating patient care and is an essential factor in patient satisfaction [65].

The second domain of ENPSS-21 is “hospitality”. The nurse’s concept of compassion and interpersonal relationships is an important element in understanding patient. The hospitality domain also included items, such as courtesy and personal appearance. In Japan, courtesy and personal appearance strongly influence patient satisfaction [66,67], and we consider these cultural factors unique to Japan.

The third domain of ENPSS-21 is “teamwork”. Nurses are part of the healthcare team and are expected to collaborate with other healthcare professionals involved in patient care [63]. Moreover, nurses have an important task to fulfill as intermediaries between multiple healthcare professions. Organizational teamwork has been reported to be associated with satisfaction, and nurses need to practice in the best interest of the patient [68]. Therefore, it is considered a very important perspective for ER nurses to collaborate with ER physicians and co-medical staff to provide medical care. 

The last domain of ENPSS-21 is “symptom management”. Patients in the ER have a wide range of distress. Appropriate analgesia affects patient satisfaction [69]. The absence of physical pain increased patient satisfaction with nursing care [70]. Therefore, distress relief is considered by some patients to be equivalent to good nursing care [71]. Namely, symptom management is considered an indispensable item for providing care that is consistent with the needs of ER patients.

Moreover, this patient satisfaction scale was developed during the COIVD-19 pandemic. During the COVID-19 pandemic, healthcare professionals have reported that adequate infection control measures are important for reducing the risk of viral infection and patient anxiety about the virus [72]. The questionnaire items used in this study included items related to infection control among nurses, which may include an important domain of recent emergency care. In addition, the compassion and interpersonal relationships of nurses are important factors for patient satisfaction [25,73,74].

Cronbach’s alpha for all domains of the ENPSS-21 scale was greater than 0.8. It was found that the ENPSS-21 had similar values to previous patient satisfaction scales, which were verified for internal consistency [59,75]. This means that each factor showed appropriate homogeneity.

Of all the hypotheses used for construct validity, the “EQ-5D-5” and “satisfaction with the response of ER physicians” were highly correlated with each of the four domains of the ENPSS-21 and summary score. Previous reports have shown an association between treatment satisfaction and QOL [55,58]. Patients with anxiety were also reported to be less satisfied with their healthcare [76], consistent with the results of this scale. In addition, satisfaction with physicians is related to overall satisfaction with healthcare [74], consistent with the hypothesis validation in this study. In contrast, a high ENPSS-21 score was not associated with the intensity of distress at the time of the emergency room visit. Although this was low compared to the intensity of distress at the emergency room visit in this study [71], it is consistent with the reported finding that VAS pain scores in the ER do not correlate with patient satisfaction [77].

## 5. Limitations

The current study has several limitations. First, test–retest reliability was not validated in this study. Having good test–retest reliability implies internal consistency of the test and ensures that the measurements obtained are representative [42]. In the future, a test–retest should be conducted to confirm reliability. Second, there are concerns about the time when data collection took place. In this study, data were collected during the COVID-19 pandemic period. As a result, the ER system was probably different from normal, which could have affected the assessment of patients. Third, this study used a minimum sample size of 100 for factor analysis from previous studies [37,42]. However, for CFA, a minimum sample of 150 is required [78]. This study did not meet that requirement, thus limiting the results of the analysis. In the future, it will be an issue to refine the items of the scale while taking the sample size into consideration.

## 6. Implications for Clinical Practice

ENPSS-21 is a brief, reliable, and valid instrument that can obtain information about patient satisfaction with ER nursing. The instrument has direct clinical utility for improving the quality of nursing care in the ER in Japan by providing a patient-centered perspective on satisfaction. The ENPSS-21 also helps address the weaknesses of the organization by measuring satisfaction regularly and comparing patient satisfaction levels relative to each other.

## 7. Conclusions

This study found that the ENPSS-21 was a robust measure of patient satisfaction, suggesting that it is possible to measure satisfaction with ER nursing. The ENPSS-21 is designed to focus on the nursing perspective of the ER. In creating the items, the ENPSS was designed to represent the emergency nursing care sought by patients on the basis of an extensive literature search and content validity. The ENPSS-21 consists of 21 items in four domains. It showed the highest correlation with the EQ-5D-5L and satisfaction with the response of ER physicians in criterion-related validity. By measuring patient-centered perspectives of satisfaction with ER nursing, factors lacking in each organization’s ER can be identified and addressed to improve the quality of nursing care in the ER.

## Figures and Tables

**Table 1:** Participant sociodemographic and clinical characteristics (*n* = 127).

Characteristic	*n* (%)
Age means (SD)	60.2 (16.4)
Sex	
Male, *n* (%)	63 (49.6)
Female, *n* (%)	64 (50.4)
Employment situation	
Unemployed, *n* (%)	43 (33.9)
Working, *n* (%)	84 (66.1)
Underlying disease, *n* (%)	
Cardiovascular disease	24 (30.0)
Respiratory tract disease	19 (23.8)
Gastrointestinal tract disease	10 (12.5)
Cancer	12 (15.0)
Diabetes	11 (13.4)
Others	21 (26.3)
None	47 (37.0)
Number of past ER visits, *n* (%)	
First time	51 (40.2)
Two times	45 (35.4)
Three times	21 (16.5)
Four times	5 (3.9)
Five times	4 (3.1)
Six times	1 (0.8)
Time of visit to the ER, *n* (%)	
Daytime (9:00–17:00)	66 (52.0)
Nighttime (17:00–9:00)	61 (48.0)

ER, emergency room; SD, standard deviation.

**Table 2:** Individual items and their factor resolutions in a 21-item patient satisfaction questionnaire regarding ER nursing.

ENPSS-21 Factor	Items	Factor 1	Factor 2	Factor 3	Factor 4
Factor 1: explanation and response (7 items)	The nurse explained everything to me before every procedure	0.973	−0.081	−0.077	−0.007
	The nurse explained to me the schedule of tests and procedures in the ER	0.839	0.078	0.073	−0.068
	The nurse explained my condition to me in terms I could understand	0.743	−0.04	−0.054	0.135
	The nurse responded appropriately to my requests	0.582	−0.025	0.39	−0.056
	The nurse gave me a chance to ask questions	0.539	0.251	−0.008	0.081
	The nurse understood my symptoms	0.389	0.246	0.127	0.099
	The nurse took great care of my privacy	0.38	0.024	0.319	0.19
Factor 2: hospitality (6 items)	The nurse treated me with care, respect, and compassion	−0.192	0.765	0.282	−0.168
	The nurse alleviated my anxiety and concerns	0.27	0.649	−0.232	0.127
	The attention to the personal appearance of the nurses was adequate	−0.162	0.605	0.22	0.193
	The nurse’s language was appropriate	0.309	0.566	−0.048	−0.12
	The nurse listened to me fully about my symptoms	0.198	0.523	0.218	−0.169
	The nurse interviewed me in detail (medical history, medications, allergy history, chief complaint, history before the visit)	0.22	0.504	−0.173	0.248
Factor 3: teamwork (3 items)	The collaboration between the nurse and the physician appeared to be smooth	−0.097	0.105	0.89	0.013
	The teamwork among the nurses appeared to be good	0.014	0.037	0.67	0.084
	The infection control measures taken by the nurses were appropriate	0.242	0.042	0.431	0.239
Factor 4: symptom management (4 items)	The nurse performed my procedure smoothly	0.024	−0.021	−0.025	0.877
	The nurse quickly responded when I was in a lot of pain	0.09	−0.16	0.185	0.828
	The nurse assessed my symptoms appropriately and performed the procedure	−0.036	0.425	0.114	0.476
	The nurse performed my procedure in a safe way	0.053	0.017	0.109	0.392

**Table 3:** Internal consistency reliability of ENPSS-21 domains.

ENPSS-21 Domain	Mean (SD)	Cronbach’s Alpha
Explanation and response	4.1 (0.6)	0.89
Hospitality	4.1 (0.6)	0.88
Teamwork	4.2 (0.5)	0.81
Symptom management	4.0 (0.6)	0.88

ENPSS-21, 21-item emergency nursing-care patient satisfaction scale; SD, standard deviation.

**Table 4:** Multitrait–multimethod correlations matrix.

Instrument	ENPSS-21 Domain Score				
	Summary Score	Explanation and Response	Hospitality	Teamwork	Symptom Management
EQ-5D-5L VAS	0.68	0.65	0.62	0.59	0.61
EQ-5D-Anxiety	0.65	0.62	0.62	0.55	0.56
Confidence in the ER physicians	0.65	0.58	0.62	0.63	0.56
Satisfaction with the response of ER physicians	0.68	0.66	0.63	0.57	0.58
Intensity of distress at the time of ER visit	−0.27	−0.29	−0.25	−0.24	−0.20
Satisfaction with the outcome of treatment in the ER	0.62	0.62	0.55	0.51	0.56

ENPSS-21, 21-item emergency nursing-care patient satisfaction scale; ER, emergency room; VAS, visual analog scale.

## Data Availability

All data from this research have been included within the manuscript.

## Appendix A

**Table A1:** Characteristics of expert panel, *n* = 19.

Characteristic	*n*
Certified nurses in emergency nursing, *n* = 10	
Sex, male	3
Age, mean (SD)	40.6 (4.9)
Work experience in emergency care	13.5 (3.2)
Healthy individuals who had visited an ER, *n* = 9	
Sex, male	3
Age, mean (SD)	42.8 (9.0)

ER, emergency room; SD, standard deviation.

## Appendix B. Nursing Emergency-Care Patient Satisfaction Scale—21 (ENPSS-21) English Version

The following questionnaire asks about the patient level of satisfaction with emergency room nurses. The goal of this questionnaire is to measure your level of satisfaction or dissatisfaction with the nurses on the basis of what you currently feel. Please place a check mark in the appropriate box for each question.

The following 20 questions relate to satisfaction with emergency room nurses.

1. The nurse explained everything to me before every procedure.
  □  Strongly disagree
  □  Slightly disagree
  □  Neutral
  □  Slightly agree
  □  Strongly agree
  □  Not applicable
2. The nurse explained the schedule of tests and procedures in the emergency room to me.
  □  Strongly disagree
  □  Slightly disagree
  □  Neutral
  □  Slightly agree
  □  Strongly agree
  □  Not applicable
3. The nurse explained my condition to me in terms I could understand.
  □  Strongly disagree
  □  Slightly disagree
  □  Neutral
  □  Slightly agree
  □  Strongly agree
  □  Not applicable
4. The nurse responded appropriately to my requests.
  □  Strongly disagree
  □  Slightly disagree
  □  Neutral
  □  Slightly agree
  □  Strongly agree
  □  Not applicable
5. The nurse gave me a chance to ask questions.
  □  Strongly disagree
  □  Slightly disagree
  □  Neutral
  □  Slightly agree
  □  Strongly agree
  □  Not applicable
6. The nurse understood my symptoms.
  □  Strongly disagree
  □  Slightly disagree
  □  Neutral
  □  Slightly agree
  □  Strongly agree
  □  Not applicable
7. The nurse took great care of my privacy.
  □  Strongly disagree
  □  Slightly disagree
  □  Neutral
  □  Slightly agree
  □  Strongly agree
  □  Not applicable
8. The nurse treated me with care, respect, and compassion.
  □  Strongly disagree
  □  Slightly disagree
  □  Neutral
  □  Slightly agree
  □  Strongly agree
  □  Not applicable
9. The nurse alleviated my anxiety and concerns.
  □  Strongly disagree
  □  Slightly disagree
  □  Neutral
  □  Slightly agree
  □  Strongly agree
  □  Not applicable
10. The attention to the personal appearance of the nurses was adequate.
  □  Strongly disagree
  □  Slightly disagree
  □  Neutral
  □  Slightly agree
  □  Strongly agree
  □  Not applicable
11. The nurse’s language was appropriate.
  □  Strongly disagree
  □  Slightly disagree
  □  Neutral
  □  Slightly agree
  □  Strongly agree
  □  Not applicable
12. The nurse listened to me fully about my symptoms.
  □  Strongly disagree
  □  Slightly disagree
  □  Neutral
  □  Slightly agree
  □  Strongly agree
  □  Not applicable
13. The nurse interviewed me in detail (medical history, medications, allergy history, chief complaint, and medical history before the visit).
  □  Strongly disagree
  □  Slightly disagree
  □  Neutral
  □  Slightly agree
  □  Strongly agree
  □  Not applicable
14. The collaboration between the nurse and the physician appeared to be smooth.
  □  Strongly disagree
  □  Slightly disagree
  □  Neutral
  □  Slightly agree
  □  Strongly agree
  □  Not applicable
15. The teamwork among the nurses appeared to be good.
  □  Strongly disagree
  □  Slightly disagree
  □  Neutral
  □  Slightly agree
  □  Strongly agree
  □  Not applicable
16. The infection control measures taken by the nurses were appropriate.
  □  Strongly disagree
  □  Slightly disagree
  □  Neutral
  □  Slightly agree
  □  Strongly agree
  □  Not applicable
17. The nurse performed my procedure smoothly.
  □  Strongly disagree
  □  Slightly disagree
  □  Neutral
  □  Slightly agree
  □  Strongly agree
  □  Not applicable
18. The nurse quickly responded when I was in a lot of pain.
  □  Strongly disagree
  □  Slightly disagree
  □  Neutral
  □  Slightly agree
  □  Strongly agree
  □  Not applicable
19. The nurse assessed my symptoms appropriately and performed the procedure.
  □  Strongly disagree
  □  Slightly disagree
  □  Neutral
  □  Slightly agree
  □  Strongly agree
  □  Not applicable
20. The nurse safely performed my procedure.
  □  Strongly disagree
  □  Slightly disagree
  □  Neutral
  □  Slightly agree
  □  Strongly agree
  □  Not applicable
  The following question is about your overall satisfaction with emergency room nurses.
  Please place a check mark in the appropriate box.
21. I felt that the overall quality of nursing care I received in the emergency room was good.
  □  Strongly disagree
  □  Slightly disagree
  □  Neutral
  □  Slightly agree
  □  Strongly agree
  □  Not applicable

## Appendix C. Nursing Emergency-Care Patient Satisfaction Scale—21 (ENPSS-21) Japanese Version

以下のアンケートは、救急外来の看護師に対するあなたの満足度を調査するものです。このアンケートの目的は、あなたが現在感じている、看護師に対する満足感を測定することです。 各質問について、該当するボックスにチェックマークを入れてください。

以下の20の質問は、救急外来の看護師に対する満足度に関するものです。

1. 看護師は全ての処置の前に説明してくれましたか。
  □  全くそう思わない
  □  そう思わない
  □  どちらでもない
  □  そう思う
  □  とてもそう思う
  □  該当なし
2. 看護師は、救急外来での検査や処置の予定について説明しましたか。
  □  全くそう思わない
  □  そう思わない
  □  どちらでもない
  □  そう思う
  □  とてもそう思う
  □  該当なし
3. 看護師は、あなたの病状に関して理解できる言葉で説明しましたか。
  □  全くそう思わない
  □  そう思わない
  □  どちらでもない
  □  そう思う
  □  とてもそう思う
  □  該当なし
4. 看護師は、あなたの要望に適切に対応しましたか。
  □  全くそう思わない
  □  そう思わない
  □  どちらでもない
  □  そう思う
  □  とてもそう思う
  □  該当なし
5. 看護師は、あなたに質問する機会を与えてくれましたか。
  □  全くそう思わない
  □  そう思わない
  □  どちらでもない
  □  そう思う
  □  とてもそう思う
  □  該当なし
6. 看護師は、あなたの症状を理解していましたか。
  □  全くそう思わない
  □  そう思わない
  □  どちらでもない
  □  そう思う
  □  とてもそう思う
  □  該当なし
7. 看護師は、あなたのプライバシーに対して十分に考慮しましたか。
  □  全くそう思わない
  □  そう思わない
  □  どちらでもない
  □  そう思う
  □  とてもそう思う
  □  該当なし
8. 看護師は、あなたに丁寧かつ敬意や思いやりのある対応をしましたか。
  □  全くそう思わない
  □  そう思わない
  □  どちらでもない
  □  そう思う
  □  とてもそう思う
  □  該当なし
9. 看護師によって、あなたの不安や心配事は軽減されましたか。
  □  全くそう思わない
  □  そう思わない
  □  どちらでもない
  □  そう思う
  □  とてもそう思う
  □  該当なし
10. 看護師の身だしなみへの配慮は十分でしたか。
  □  全くそう思わない
  □  そう思わない
  □  どちらでもない
  □  そう思う
  □  とてもそう思う
  □  該当なし
11. 看護師の言葉遣いは適切でしたか。
  □  全くそう思わない
  □  そう思わない
  □  どちらでもない
  □  そう思う
  □  とてもそう思う
  □  該当なし
12. 看護師は、あなたの症状について十分に話を聞いていましたか。
  □  全くそう思わない
  □  そう思わない
  □  どちらでもない
  □  そう思う
  □  とてもそう思う
  □  該当なし
13. 看護師は、あなたに対して詳しく問診（既往歴、内服薬、アレルギー歴、主訴、受診前の経緯などの聴取）をしていましたか。
  □  全くそう思わない
  □  そう思わない
  □  どちらでもない
  □  そう思う
  □  とてもそう思う
  □  該当なし
14. 看護師と医師との連携はスムーズでしたか。
  □  全くそう思わない
  □  そう思わない
  □  どちらでもない
  □  そう思う
  □  とてもそう思う
  □  該当なし
15. 看護師同士のチームワークは良かったですか。
  □  全くそう思わない
  □  そう思わない
  □  どちらでもない
  □  そう思う
  □  とてもそう思う
  □  該当なし
16. 看護師の感染対策は十分と感じましたか。
  □  全くそう思わない
  □  そう思わない
  □  どちらでもない
  □  そう思う
  □  とてもそう思う
  □  該当なし
17. 看護師は、あなたの処置をスムーズに行っていましたか。
  □  全くそう思わない
  □  そう思わない
  □  どちらでもない
  □  そう思う
  □  とてもそう思う
  □  該当なし
18. 看護師は、あなたの苦痛が強いとき迅速に対応してくれましたか。
  □  全くそう思わない
  □  そう思わない
  □  どちらでもない
  □  そう思う
  □  とてもそう思う
  □  該当なし
19. 看護師は、あなたの症状を適切に判断して処置を行なっていましたか。
  □  全くそう思わない
  □  そう思わない
  □  どちらでもない
  □  そう思う
  □  とてもそう思う
  □  該当なし
20. 看護師は、処置を行う際、安全に配慮していましたか。
  □  全くそう思わない
  □  そう思わない
  □  どちらでもない
  □  そう思う
  □  とてもそう思う
  □  該当なし
  次の質問は、救急外来の看護師に対するあなたの総合的な満足度についてです。
  該当するボックスにチェックマークを入れてください。
21. 救急外来受診中に受けた看護ケアの全体的な質は良いと感じましたか。
  □  全くそう思わない
  □  そう思わない
  □  どちらでもない
  □  そう思う
  □  とてもそう思う
  □  該当なし
